# Does the dose make the poison? Neurotoxic insecticides impair predator orientation and reproduction even at low concentrations

**DOI:** 10.1002/ps.6789

**Published:** 2022-01-20

**Authors:** Luis C Passos, Michele Ricupero, Antonio Gugliuzzo, Marianne A Soares, Nicolas Desneux, Geraldo A Carvalho, Lucia Zappalà, Antonio Biondi

**Affiliations:** ^1^ Laboratório de Ecotoxicologia e MIP, Departamento de Entomologia Universidade Federal de Lavras Lavras Brazil; ^2^ Department of Agriculture, Food and Environment University of Catania Catania Italy; ^3^ Université Côte d'Azur, INRAE, CNRS, UMR ISA Nice 06000 France

**Keywords:** ecotoxicology, sublethal effects, predatory mirid, pesticides, integrated pest management

## Abstract

**BACKGROUND:**

Pesticides can be noxious to non‐target beneficial arthropods and their negative effects have been recently recognized even at low doses. The predator *Nesidiocoris tenuis* (Reuter) (Hemiptera: Miridae) plays an important role in controlling insect pests in solanaceous crops, but its concurrent herbivory often poses relevant concerns for tomato production. Although insecticide side effects on *N. tenuis* have been previously studied, little is known on the potential implications of neurotoxic chemicals at low concentrations. We assessed the baseline toxicity of three neurotoxic insecticides (lambda‐cyhalothrin, spinosad and chlorpyrifos) on *N. tenuis* by topical contact exposure. The behavioral and reproduction capacity of the predator was then investigated upon exposure to three estimated low‐lethal concentrations (LC_1_, LC_10_ and LC_30_).

**RESULTS:**

Predator survival varied among insecticides and concentrations, with LC_30_/label rate ratios ranging from 8.45% to 65.40% for spinosad and lambda‐cyhalothrin, respectively. All insecticides reduced the fertility of *N. tenuis* females at all estimated low‐lethal concentrations. Chlorpyrifos seriously compromised predator orientation towards a host plant even at LC_1_, while the same effect was observed for lambda‐cyhalothrin and spinosad solely at LC_30_. Lambda‐cyhalothrin (at all concentrations) and chlorpyrifos (at LC_10_ and LC_30_) also affected the time taken by *N. tenuis* females to make a choice.

**CONCLUSION:**

The results indicate that all three insecticides can be detrimental to *N. tenuis* and should be avoided when presence of the predator is desirable. © 2022 The Authors. *Pest Management Science* published by John Wiley & Sons Ltd on behalf of Society of Chemical Industry.

## Graphical abstract

Neurotoxic insecticides negatively affected the behavior and reproduction of the predator *Nesidiocoris tenuis*, even at low concentrations. Our study suggest that these compounds should be avoided when the presence of the predator is desirable.

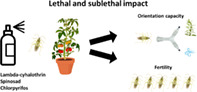

## 1 INTRODUCTION

Pesticides have been incriminated for their negative consequences on biodiversity and its functioning, although their relevance in controlling plant pests effectively remains undeniable.^1^, ^2^ Pesticides can potentially affect non‐target organisms present in the agroecosystem, leading to disruption of the ecological services they provide, such as pollination, nutrient cycling and biological control.^3^, ^4^ For this reason, studies on the side effects of pesticides are encouraged to provide new insights to mitigate their negative impacts on non‐target beneficial arthropods.^5^, ^6^, ^7^, ^8^, ^9^ This is especially relevant in integrated pest management (IPM) programs in which natural enemies are often deliberately released and/or conserved to reduce pest populations.^10^, ^11^, ^12^, ^13^, ^14^

Ecotoxicological screenings are usually based on guidelines developed by non‐governmental institutions, and in the European Union (EU) the ecotoxicological risk assessment of pesticides towards non‐target arthropods was developed in the Guidance Document on Terrestrial Ecotoxicology,^15^ following the recommendation of the European standard characteristics of beneficials regulatory testing (ESCORT) of the Society of environmental toxicology and chemistry (SETAC) for non‐bee arthropods.^16^, ^17^ Most ecotoxicology studies consist of laboratory trials aimed at testing the highest pesticide dose recommended by manufacturers. However, pesticides are naturally degraded by biotic and abiotic factors,^18^, ^19^ and their drift may also occur in the field resulting in lower doses compared with their initial application.^20^, ^21^ Therefore, non‐target organisms present in the agroecosystem can be exposed to chemical residues at low concentrations and, surviving individuals may experience related sublethal effects.^19^, ^22^, ^23^, ^24^ These effects may include lower fertility and a reduction in predation/parasitism ability, which can negatively affect the establishment of natural enemies in the field and bias their efficiency in reducing pest populations.^3^, ^25^, ^26^

Hemipteran predators are of paramount importance for the biological control of insect pests in greenhouse crops because they are able to control populations of several arthropod pests.^26^, ^27^, ^28^, ^29^, ^30^, ^31^Among mirid predators (Hemiptera: Miridae), the zoophytophagous *Nesidiocoris tenuis* (Reuter) (Hemiptera: Miridae) is one of the most used species for biological control in the Palaearctic. *Nesidiocoris tenuis* has a multifaceted role for greenhouse pest control due to its high efficacy against a number of pests including aphids, whiteflies and lepidopterans, such as the South American tomato pinworm, *Tuta absoluta* (Meyrick) (Lepidoptera: Gelechiidae).^32^ Moreover, the use of *N. tenuis* has been fostered because of its ability in priming induced plant defense mechanisms.^33^, ^34^, ^35^, ^36^, ^37^, ^38^ However, owing to its plant‐feeding activity when prey is scarce, *N. tenuis* can become a pest because this predator can cause plant damage at high population levels.^39^, ^40^, ^41^ Despite this drawback, IPM programs still rely on the biological control provided by *N. tenuis*. Therefore, the predator can be often exposed to organic and/or synthetic insecticides routinely adopted in these programs.^42^, ^43^, ^44^, ^45^

Earlier studies investigated the impact of insecticides on *N. tenuis* in terms of lethal and sublethal effects, by exposing *N. tenuis* adults to synthetic and organic neurotoxic compounds via different exposure routes (i.e., contaminated prey, direct spray and residual contact).^27^, ^28^, ^46^, ^47^ Nevertheless, most studies investigated only the maximum label rate of these compounds. To the best of our knowledge, there is no information regarding the effects of low insecticide concentrations on *N. tenuis* orientation capacity, which may ultimately affect the success of this predator as a biological control agent.

In this study, we hypothesized that low concentrations of neurotoxic insecticides might have detrimental effects on the physiology and behavior of *N. tenuis*. We tested this hypothesis through laboratory trials aiming to assess the fertility and olfactory response of *N. tenuis* adults topically exposed to three low‐lethal concentrations (LC_30_, LC_10_, LC_1_) of insecticides, previously estimated for this mirid predator. Our findings may help in understanding the convolutions of pesticide side effects at low concentrations on natural enemies and provide new useful insights into the association between the predator *N. tenuis* and chemical insecticides in pest control.

## 2 MATERIALS AND METHODS

### 2.1 Biological materials

*Nesidiocoris tenuis* for laboratory rearing were obtained from periodic collections in organic open tomato greenhouses located in Fiumefreddo (Catania, Italy). Collected specimens were morphologically identified and reared in the laboratory as follows. Briefly, adults of *N. tenuis* (~150 individuals) were kept in entomological cages (32 × 40 × 70 cm) covered by fine net mesh and containing pesticide‐free sesame (*Sesamum indicum* L., variety T‐85 Humera) potted seedlings (~30 cm in height), as water and oviposition sources, according to the methodology described by Biondi *et al*.^48^ The commercial mixture of the alternative prey *Ephestia kuehniella* Zeller (Lepidoptera: Pyralidae) eggs and *Artemia* spp. cysts (i.e., Entofood® Koppert) was offered *ad libitum* to the predators as an additional food source. *Nesidiocoris tenuis* adults were kept on sesame plants for 3 days to allow mating and oviposition events; subsequently, *N. tenuis* adults were collected with a mechanical aspirator and transferred to new cages as described above. Sesame plants bearing *N. tenuis* eggs were isolated inside the cages for egg hatching and the development of newly hatched nymphs to adulthood. Half of the newly molted *N. tenuis* adults were collected with a mechanical aspirator and used for the bioassays, whereas the remainder were added to the rearing. New sesame plants and Entofood® were added to each cage twice a week. The rearing was maintained under laboratory conditions (25 ± 1°C, 55% ± 5% relative humidity, and a 14:10 h light/dark photoperiod) at the Department of Agriculture, Food and Environment of the University of Catania (Italy).

### 2.2 Insecticides

To assess the potential physiological and behavioral effects on *N. tenuis*, three neurotoxic insecticides were evaluated in this study. The insecticides, followed by their tradename, manufacturer, chemical group and mode of action, were: lambda‐cyhalothrin (Karate Zeon®, Syngenta Italia S.p.a.), a pyrethroid, Na^+^ channel modulator; spinosad (Laser®, Dow AgroSciences S.r.l.), a spinosyn, nicotinic acetylcholine receptor allosteric modulator; and chlorpyrifos (Dursban®, Dow AgroSciences S.r.l.), an organophosphate, acetylcholinesterase (AChE) inhibitor. Lambda‐cyhalothrin and chlorpyrifos are both synthetic insecticides used in conventional tomato crops in many countries, whereas spinosad is a naturally derived insecticide, therefore its use is allowed in both conventional and organic crops. These insecticides were selected due to their potential use in tomato crops to control hemipteran and lepidopteran pests (such as aphids, whiteflies and *T. absoluta*), which are also *N. tenuis* prey.

### 2.3 Insecticides baseline toxicity toward *Nesidiocoris tenuis*

In this bioassay, we assessed the concentration–mortality response relationship of *N. tenuis* adult stage to lambda‐cyhalothrin, spinosad and chlorpyrifos by topical contact exposure. Newly emerged females (~2 days old) were exposed by topical spray to different concentrations of the insecticides. For each insecticide, six or seven concentrations, including of the highest label rate, were tested (see Table 1). Stock solutions were prepared with dilution of insecticidal formulations in distilled water, according to the manufacturer's recommendations. In addition, an untreated control with distilled water was included for all the insecticides (referred to as “zero concentration”). The insecticide dilutions were based on preliminary observations aimed at identifying the minimum dose needed to cause 100% mortality of *N. tenuis* females and the maximum dose that does not significantly affect the mortality of the treated insects in comparison with the untreated control.

**TABLE 1:** Baseline toxicity of three insecticides toward *Nesidiocoris tenuis* females 48 h after topical contact exposure by spraying

Insecticide	Tradename	% a.i.	Label rate (ppm)	Slope ± SE	χ^2^ (*df*)	*p*‐value	Lethal concentration (ppm)	95% Confidence limits (ppm)	% LC/LR^a^
Spinosad	Laser®	44.20	0.3315	1.974 ± 0.260	33.355 (31)	0.353	LC_1_ = 3.37 × 10^−3^	1.35 × 10^−3^ to 5.88 × 10^−3^	1.08
							LC_10_ = 1.14 × 10^−2^	6.71 × 10^−3^ to 1.62 × 10^−2^	3.32
							LC_30_ = 2.75 × 10^−2^	2.00 × 10^−2^ to 3.60 × 10^−2^	8.44
Lambda‐cyhalothrin	Karate Zeon®	9.48	0.0236	1.301 ± 0.201	42.901 (36)	0.201	LC_1_ = 6.39 × 10^−4^	1.10 × 10^−4^ to 1.68 × 10^−3^	2.70
							LC_10_ = 4.06 × 10^−3^	1.49 × 10^−3^ to 7.33 × 10^−3^	17.13
							LC_30_ = 1.55 × 10^−2^	9.24 × 10^−3^ to 2.22 ∙ 10^−2^	65.40
Chlorpyrifos	Dursban®	44.53	0.3340	0.948 ± 0.202	35.563 (33)	0.349	LC_1_ = 8.87 × 10^−4^	2.30 × 10^−4^ to 4.09 × 10^−4^	0.27
							LC_10_ = 1.12 × 10^−2^	1.72 × 10^−3^ to 2.58 × 10^−2^	3.35
							LC_30_ = 7.05 × 10^−2^	3.34 × 10^−2^ to 1.14 × 10^−1^	21.11

^a^ % LC/LR is percentage of the estimated low‐lethal concentration in comparison with the highest label rate recommended in tomato crop.

An adapted methodology for insecticide topical contact application on *N. tenuis* adult stage was developed. Briefly, five *N. tenuis* females were isolated together in conical ventilated plastic tubes (Falcon®, 50 ml) and maintained at low temperature inside an insulated thermic box with ice packs for 3 h to reduce insect mobility. Thereafter, each group of *N. tenuis* females was placed in a plastic cup (100 ml) and topically sprayed with insecticide solutions using a hand‐sprayer (50 ml). The inside of the plastic cups was covered by absorbent paper to prevent the formation of insecticide droplets in the arena, preventing insect mortality via drowning. Clean and new absorbent paper was changed in each replicate for every insecticide–concentration combination. After spraying, each group of five *N. tenuis* females was transferred to an acrylic ventilated pot (5.5 cm in diameter × 3 cm height), along with a zucchini (*Cucurbita pepo* L.) leaf disc and Entofood®. Each pot containing five females was considered one replicate. Mortality caused by the insecticides on *N. tenuis* females was evaluated after 48 h. Eight replicates were performed for each insecticide–concentration combination.

### 2.4 Sublethal effects of insecticides on *Nesidiocoris tenuis* fertility

The aim of this bioassay was to evaluate whether low concentrations of lambda‐cyhalothrin, spinosad and chlorpyrifos could affect the fertility of the predator *N. tenuis*. Based on the results of the previous bioassay, newly molted *N. tenuis* males and females (2 days old) from the rearing were exposed to three low‐lethal concentrations (LC_1_, LC_10_ and LC_30_) of the aforementioned insecticides. These concentrations were chosen to expose the predators to low concentrations that can occur under field conditions after environmental degradation of a full label spray, including a lethal range from very low mortality (LC_1_) to moderate mortality (LC_30_).

Adult females (2 days old) were sprayed with the low‐lethal concentrations mentioned above and distilled water, as described in Section 2.3. Sprayed couples were kept in a ventilated plastic cup (400 ml) containing a green bean pod (*Phaseolus vulgaris* L., cv. ‘Garrafal enana’) as a water source and oviposition substrate,^28^, ^49^ and *E. kuehniella* eggs (1 g) as food supply in the arena. Each *N. tenuis* couple was kept in the aforementioned arena for 3 days to increase mating success and let the females oviposit into the bean. The experimental arenas containing green bean pods with *N. tenuis* eggs were maintained under laboratory conditions as described above, and the number of newly emerged nymphs was recorded daily under a stereomicroscope and removed with a soft paintbrush. The evaluation was conducted for 20 days until no new nymph emerged. For each pesticide–concentration combination and the control, the fertility of 25 *N. tenuis* couples (i.e., 25 replicates) was evaluated.

### 2.5 Sublethal effects of insecticides on *Nesidiocoris tenuis* orientation

The aim of this bioassay was to evaluate whether the orientation of the predator *N. tenuis* could be affected by the three low‐lethal concentrations (LC_1_, LC_10_ and LC_30_) of lambda‐cyhalothrin, spinosad and chlorpyrifos. Adult females (2 days old) were sprayed with the low‐lethal concentrations mentioned above and distilled water, as described in Section 2.3. After being sprayed topically, *N. tenuis* females were starved for 24 h in transparent vials (1.5 cm in diameter × 6 cm height) with a wet cotton wad as the only water source. Thereafter, each *N. tenuis* female was transferred in a two‐way olfactometer (main arm and lateral arms 15 cm long and 4 cm internal diameter). The odor sources used were clean air and a sesame plant (~20 cm height). Sesame plant was chosen because previous studies demonstrated that this plant is highly attractive to *N. tenuis*.^48^, ^50^ A sesame plant was placed inside one of the cylindrical glass jars (5 L volume) connected to the lateral arms of the olfactometer. An air pump (Airfizz®, Ferplast) produced a unidirectional flow (150 ml min^−1^) that passed through a water filter before entering the olfactometer system, conducting the air through the olfactometer lateral arms and reaching thus the main arm. The olfactometer was placed vertically on the bench surface and *N. tenuis* females were placed individually on the central arm. The bioassays were performed in a dark room, with controlled environmental conditions (25 ± 1°C, 60 ± 10% R. H.) and were conducted between 9:00 a.m. and 6:00 p.m. The olfactometer was illuminated by 22 W cool‐white fluorescent lamps, positioned 80 cm above the olfactometer arms, according to Naselli *et al*.^50^

The choice of each female was considered when it crossed half of the lateral arm. Each predator was observed for 5 min and, if no choice was made after that time, non‐responder *N. tenuis* females were discarded from the data set. After every two tested females, the olfactometer was inverted to reduce environmental interference in the insect response. For each insecticide–concentration combination, at least 30 replicates, each composed of an insect that have did a choice, were carried out. The time taken for *N. tenuis* females to make a choice (for insects that made a choice) was also recorded.

### 2.6 Statistical analyses

The baseline toxicity of lambda‐cyhalothrin, spinosad and chlorpyrifos on *N. tenuis* by topical contact exposure was carried out through a log‐probit regression model.^51^ The preference data of *N. tenuis* towards sesame plants were analyzed using a chi‐squared goodness‐of‐fit to determine whether the female attraction to sesame plants was different from a 50:50 distribution.

Data regarding time taken by *N. tenuis* females to make a choice and fertility were tested for normality and homoscedasticity;^52^, ^53^ however, these assumptions were not met. Therefore, these data were fitted to generalized linear models (GLMs),^54^ and potential interaction between factors (four treatments × three concentrations) was tested. The models were fitted using the Poisson family for fertility and negative binomial family for time taken by *N. tenuis* females to make a choice (Poisson and quasi‐Poisson families were first tested, but the negative binomial model presented a better fit). Means were separated by a post‐hoc Tukey's HSD test (*p* < 0.05). Probit analyses were performed in the statistical program SPSS v. 21.0 (IBM Corp.), whereas the analyses for the fertility and olfactory response bioassays were performed in R v. 3.6.0 (R Foundation for Statistical Computing), using the packages *car* and *MASS* for model fitting and the package *multcomp* to separate means.^55^, ^56^, ^57^

## 3 RESULTS

### 3.1 Insecticides baseline toxicity toward *Nesidiocoris tenuis*

The probit models were fitted to observed data for all the treatments (i.e., there were no significant differences between the observed and the expected data), validating the low‐lethal concentrations for all the tested neurotoxic insecticides (Table 1). All insects treated with distilled water only (“zero concentration”) survived throughout the evaluation period. Lambda‐cyhalothrin was the insecticide with the lowest values of LC_1_, LC_10_ and LC_30_, being the most lethal active ingredient for *N. tenuis* females. Spinosad and chlorpyrifos also presented high toxicity to the predator as highlighted by the low LC_30_ values estimated for these compounds. Nevertheless, despite lambda‐cyhalothrin being the most toxic active ingredient, it was observed that the proportion values between the estimated LC_10_ and LC_30_ and the maximum label rate were higher for this insecticide (17.13% and 65.40%) in comparison with those observed for spinosad (3.32% and 8.45%) and chlorpyrifos (3.35% and 21.11%) (Table 1).

### 3.2 Sublethal effects of insecticides on *Nesidiocoris tenuis* fertility

**FIGURE 1:**
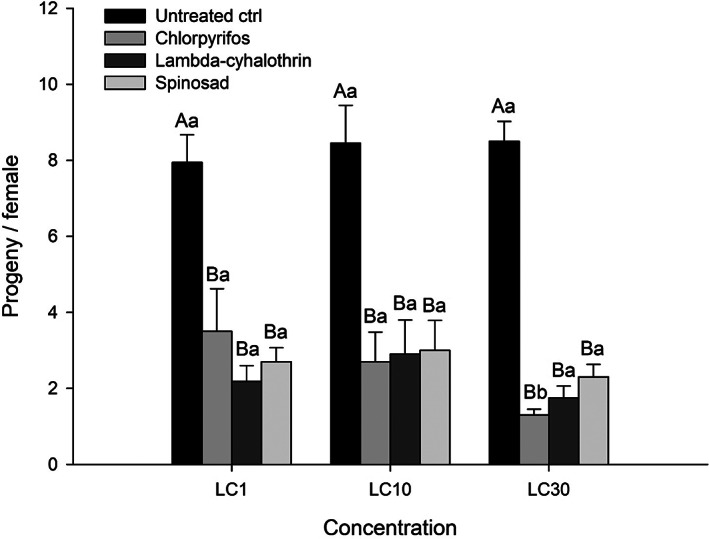
Mean (±) fertility values for *Nesidiocoris tenuis* females after topical contact exposure to three insecticides at three low‐lethal concentrations and distilled water (untreated control). Different upper case letters indicate significant differences among treatments in a concentration, whereas different lower case letters indicate significant differences in the concentrations for a treatment (GLM – Poisson distribution, Tukey's HSD test, *p* < 0.05).

Although the GLM revealed no significant insecticide × concentration interaction (χ^2^ = 12.023, *df* = 6, *p* = 0.061), all the tested insecticides significantly reduced the fertility of *N. tenuis* females at all the evaluated concentrations (LC_1_: χ^2^ = 64.642, *df* = 3, *p* < 0.001; LC_10_: χ^2^ = 73.707, *df* = 3, *p* < 0.001; LC_30_: χ^2^ = 118.560, *df* = 3, *p* < 0.001). The reduction in fertility was higher for chlorpyrifos at LC_30_ (χ^2^ = 9.939, *df* = 2, *p* = 0.007), whereas no differences were observed among the concentrations for lambda‐cyhalothrin (χ^2^ = 2.659, *df* = 2, *p* = 0.265), spinosad (χ^2^ = 1.008, *df* = 2, *p* = 0.604) and the control (χ^2^ = 0.427, *df* = 2, *p* = 0.808) (Figure 1).

### 3.3 Sublethal effects of insecticides on *Nesidiocoris tenuis* orientation

**FIGURE 2:**
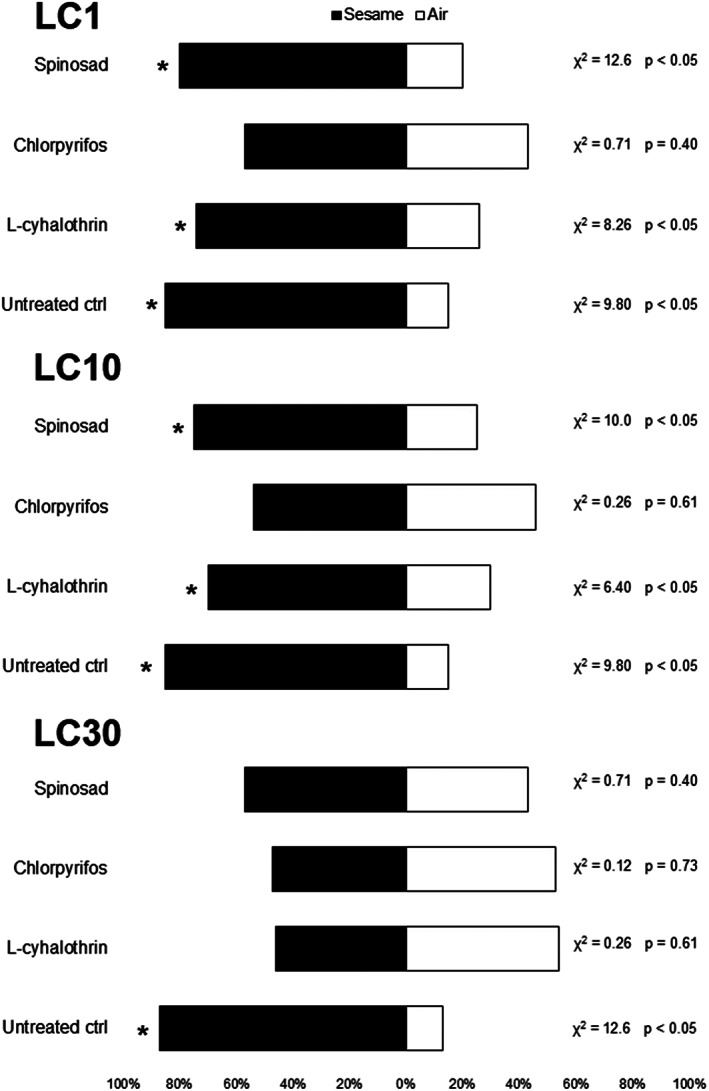
Response of *Nesidiocoris tenuis* females topically exposed to three insecticides at three low‐lethal concentrations (LC_1_, LC_10_ and LC_30_) and distilled water (untreated control) towards the volatiles produced by a *Sesamum indicum* plant. The percentages indicate the proportion of choices for sesame and clean air. Asterisks indicate differences in the attraction to *S. indicum* and clean air according to the likelihood chi‐squared (*p* < 0.05).

A significant attraction towards sesame plants was expected for insects that did not experience any insecticide exposure,^50^ and it was confirmed for all control treatments. Therefore, this was taken as a reference for the percentage of insects orienting toward sesame compared with clean air for the treatments with insecticides. The preference of *N. tenuis* females for sesame plants instead of clean air was not affected by lambda‐cyhalothrin or spinosad at LC_1_ and LC_10_. However, the choices of insects treated with chlorpyrifos did not differ between sesame and air for these two low‐lethal concentrations. At LC_30_, all insecticides affected *N. tenuis* orientation, resulting in no difference between the proportion of choices for sesame and clean air (Figure 2).

**FIGURE 3:**
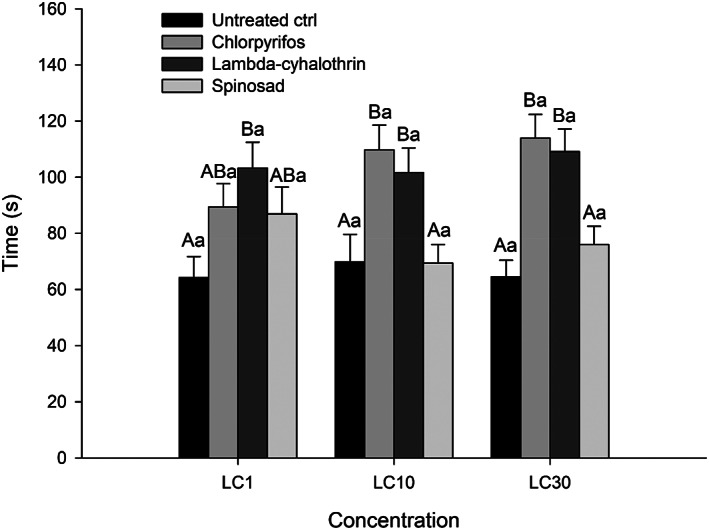
Mean (±SE) time taken (s) by *Nesidiocoris tenuis* females to make a choice between the volatiles emitted by a *Sesamum indicum* plant or clean air after topical contact exposure to three insecticides at three low‐lethal concentrations and distilled water (untreated control). Different upper case letters indicate differences among treatments in a concentration, whereas different lower case letters indicate differences in the concentrations for a treatment (GLM – Negative Binomial distribution, Tukey's HSD test, *p* < 0.05).

Differences in the time taken by *N. tenuis* females to make a choice were observed in all low‐lethal concentrations (LC_1_: χ^2^ = 9.358, *df* = 3, *p* = 0.024; LC_10_: χ^2^ = 22.566, *df* = 3, *p* < 0.001; LC_30_: χ^2^ = 33.291, *df* = 3, *p* < 0.001) (Figure 3). Insects treated with all the tested concentrations of lambda‐cyhalothrin took longer to make a choice in comparison with the control treatment. The same was observed for insects treated with chlorpyrifos at LC_10_ and LC_30_. For females treated with all concentrations of spinosad time taken to make a choice was not affected in comparison with the control treatment. No differences were observed among concentrations for any of the treatments (control: χ^2^ = 0.508, *df* = 2, *p* = 0.777; lambda‐cyhalothrin: χ^2^ = 0.634, *df* = 2, *p* = 0.729; chlorpyrifos: χ^2^ = 4.981, *df* = 2, *p* = 0.083; spinosad: χ^2^ = 3.589, *p* = 0.166) (Figure 3). There was no interaction between treatments and concentrations for the time taken by *N. tenuis* females to make a choice (χ^2^ = 9.066, *df* = 6, *p* = 0.170), therefore the data were evaluated separately.

## 4 DISCUSSION

In many systems, broad‐spectrum insecticides, such as neurotoxic insecticides, are the most used compounds for pest control because of their effectiveness in controlling pests. However, a vast literature has documented concerning detrimental effects on beneficial organisms caused by these effective tools.^3^, ^58^, ^59^ To preserve the ecological functions of beneficial organisms in the agroecosystem (including biological control) selective insecticides should be preferred in pest management.^5^, ^22^, ^26^, ^60^, ^61^, ^62^ Additionally, insecticides can cause sublethal effects that can bias the biological control provided by predators and parasitoids.^3^, ^24^ These alterations can be observed in individuals that survived both full label rates of selective compounds and lower concentrations of broad‐spectrum insecticides, which can occur after natural degradation under field conditions.^22^, ^63^, ^64^, ^65^

Probit models are often used to estimate concentration–mortality of pesticides to pests and natural enemies, in order to select efficient and safe compounds, respectively.^49^, ^65^, ^66^, ^67^, ^68^ In our observations, lambda‐cyhalothrin was the most toxic compound for the predator, because lower values were estimated for all lethal concentrations. Spinosad was the least toxic insecticide at LC_1_ towards *N. tenuis*; however, at LC_30_ this insecticide was more toxic than chlorpyrifos. The highest slope estimated for spinosad treatment indicates that a slight increase in insecticide concentration may lead to high predator mortality.^69^ Moreover, due to the lower active ingredient concentration in the lambda‐cyhalothrin based insecticide, the difference between the maximum label rate and the estimated LC_10_ and LC_30_ are lower for lambda‐cyhalothrin than for the spinosad and chlorpyrifos commercial products. Therefore, despite the higher LC_10_ and LC_30_ values observed for spinosad and chlorpyrifos, these insecticides might be even more toxic than lambda‐cyhalothrin under field conditions.

Besides mortality, all insecticides at the three evaluated concentrations reduced the fertility of the predator *N. tenuis*. Reproductive parameters are among the most sensitive biological characteristics to insecticides and the most important in terms of population dynamics.^70^ Similar to our results, a reduction in *N. tenuis* progeny was also observed for the pyrethroids cypermethrin and deltamethrin.^28^, ^46^ Additionally, pyrethroids can be used at sublethal concentrations to contaminate insect‐proof nets, and Biondi *et al*. found that the continuous exposure of *T. absoluta* adults to such nets can cause a variety of chronic sublethal effects rather than acute toxicity.^71^

Lower concentrations of the organophosphate chlorpyrifos were also frequently reported as causing negative effects on the reproduction of natural enemies. At LC_30_ several sublethal effects were observed on the hemipteran *Andrallus spinidens* Fabricius (Hemiptera: Pentatomidae), such as reduction in fertility and enzyme activity, and alterations in life table parameters.^72^ Fernandes *et al*. observed negative effects on reproduction after chlorpyrifos exposition at LC_20_ for the predator *Orius insidiosus* (Say) (Hemiptera: Anthocoridae).^67^ Moreover, spinosad reduced the offspring of the predatory bugs, such as *Orius laevigatus* (Fieber) (Hemiptera: Anthocoridae),^22^ *Macrolophus pygmaeus* (Rambur) (Hemiptera: Miridae)^27^, ^73^ and *Deraeocoris brevis* (Uhler) (Hemiptera: Miridae).^74^

Negative effects were also observed in the behavioral traits of *N. tenuis*. In the insecticide treatments, spinosad and lambda‐cyhalothrin at LC_30_ and chlorpyrifos at all concentrations affected the orientation ability of the predator. Moreover, the two synthetic insecticides also increased the time taken by *N. tenuis* females to make a choice. Because of their neurotoxic action, all three insecticides can affect the capacity of the nervous system to react to external stimuli.^59^, ^63^, ^72^, ^75^

The behavioral results in this study are consistent with neurotoxicity associated with lambda‐cyhalothrin. The deleterious effects caused by pyrethroids result from a blockage in electrical stimulus conduction as a consequence of the permanent opening of sodium channels while the insecticide is acting, leading to behavioral and physiological impacts.^59^, ^76^ Desneux *et al*. also observed that sublethal doses of lambda‐cyhalothrin (LD_0.1_) affected the orientation behavior of the parasitoid *Aphidius ervi* Haliday (Hymenoptera: Braconidae).^63^ The parasitoid *Aphidius colemani* Viereck (Hymenoptera: Braconidae) exhibited a reduction in parasitism and longevity after treatment with sublethal concentrations of lambda‐cyhalothrin.^77^ Soares *et al*. also observed alterations in *N. tenuis* behavior caused by lambda‐cyhalothrin.^26^

Similarly, a lack of coordination is associated with chlorpyrifos intoxication.^72^ The biased orientation of *N. tenuis* females treated with chlorpyrifos at LC_1_, LC_10_ and LC_30_ indicates that this insecticide can affect the predator behavior even at very low concentrations. The time taken by *N. tenuis* females to make a choice also increased after treatment with LC_10_ and LC_30_. Fernandes *et al*. observed negative effects on predation rate after chlorpyrifos exposure at LC_20_ for the predator *O. insidiosus*.^67^ The predator *M. pygmaeus* also showed behavioral alterations (reduced attack rate and increased handling time) after treatment with chlorpyrifos at LC_30_.^78^

By contrast, studies regarding the side effects of spinosad on the behavior of beneficial insects are scarce. For example, Barbosa *et al*. observed alterations in walking activity in the stingless bee *Melpona quadrifasciata* Depeleiter (Hymenoptera: Apidae) after exposure to spinosad.^79^ Nevertheless, owing to the toxic effect on the nervous system, insects intoxicated by spinosad may present symptoms like a lack of coordination, trembling of appendages and a compromised perception of external stimuli, which can ultimately result in reduced predatory capacity for a natural enemy.^75^, ^80^, ^81^, ^82^

Plants from different botanical families, such as Asteraceae, Solanaceae and Pedaliaceae, are suitable for *N. tenuis* biological development. These plants could serve as water and oviposition sources, and are also plants where *N. tenuis* prey can be found in the field.^39^, ^48^ For this reason, disrupting the predators’ capacity to locate host plants directly influences their survival and success as biological control agents. The misorientation caused by lower concentrations of insecticides could also compromise *N. tenuis* capacity to locate plants infested with herbivorous prey, as observed for the predator *Cyrtorhinus lividipennis* Reuter (Hemiptera: Miridae) after exposure to the pyrethroid deltamethrin.^83^ Further study is needed on this point of the system.

In summary, we observed that spinosad, chlorpyrifos and lambda‐cyhalothrin can be toxic to the predator *N. tenuis,* even at low concentrations, with effects on fertility and orientation. In addition to the laboratory results, field trials should be performed to confirm the toxicity of the compounds exploring different exposure routes (i.e., residual contact and ingestion of contaminated prey) and/or by testing the potential side effects toward insect parasitoids exploited in tomato crops.^13^, ^84^, ^85^

## 5 CONCLUSIONS

The baseline toxicity showed that the insecticides were toxic to *N. tenuis* females. The sublethal effects caused by the tested concentrations of the three insecticides were also relevant. Even at LC_1_ and LC_10_ the fertility of *N. tenuis* females was compromised by all the insecticides. In addition, sublethal effects on predator orientation were observed. We concluded that the three insecticides were noxious to *N. tenuis* and should be avoided when the presence of the predator is desirable. Nevertheless, field trials must be carried out to confirm their sublethal toxicity and overall risk (interaction of exposure, hazard and other factors).

Additionally, the negative effects on *N. tenuis* orientation observed in the current study provide a basis for further research aiming to elucidate how neurotoxic insecticides impair *N. tenuis* capacity to locate host plants or herbivorous prey, by investigating alterations in the gene expression of odorant‐binding and chemosensory proteins that might be involved in plant volatile reception by employing electro‐antennography and quantitative real‐time polymerase chain reaction bioassays.^86^, ^87^, ^88^ Moreover, the results highlight the importance of investigating other insecticides that might have a narrow spectrum and that would be more compatible with sustainable IPM.

## CONFLICTS OF INTEREST

The authors declare that they have no conflict of interest.

## Data Availability

The data that support the findings of this study are available from the corresponding author upon reasonable request.

## References

[1] Horowitz AR , Ghanim M , Roditakis E , Nauen R and Ishaaya I , Insecticide resistance and its management in *Bemisia tabaci* species. J Pest Sci 93:893–910 (2020).

[2] Sánchez‐Bayo F , Indirect effect of pesticides on insects and other arthropods. Toxics 9:177 (2021). 34437495 10.3390/toxics9080177 PMC8402326

[3] Desneux N , Decourtye A and Delpuech J‐M , The sublethal effects of pesticides on beneficial arthropods. Annu Rev Entomol 52:81–106 (2007). 16842032 10.1146/annurev.ento.52.110405.091440

[4] Köhler HR and Triebskorn R , Wildlife ecotoxicology of pesticides: can we track effects to the population level and beyond? Science 341:759–765 (2013). 23950533 10.1126/science.1237591

[5] Biondi A , Zappalà L , Stark JD and Desneux N , Do biopesticides affect the demographic traits of a parasitoid wasp and its biocontrol services through sublethal effects? PLoS One 8:e76548 (2013). 24098793 10.1371/journal.pone.0076548 PMC3787011

[6] Bueno ADF , Carvalho GA , Santos ACD , Sosa‐Gómez DR and Silva DMD , Pesticide selectivity to natural enemies: challenges and constraints for research and field recommendation. Cienc Rural 47:1–10 (2017).

[7] Mansour R , Belzunces LP , Suma P , Zappalà L , Mazzeo G , Grissa‐Lebdi K *et al*., Vine and citrus mealybug pest control based on synthetic chemicals. A review. Agron Sustain Dev 38:1–20 (2018).

[8] Campolo O , Puglisi I , Barbagallo RN , Cherif A , Ricupero M , Biondi A *et al*., Side effects of two citrus essential oil formulations on a generalist insect predator, plant and soil enzymatic activities. Chemosphere 257:127252 (2020). 32526470 10.1016/j.chemosphere.2020.127252

[9] Menail AH , Boutefnouchet‐Bouchema WF , Haddad N , Taning NTC , Smagghe G and Loucif‐Ayad W , Effects of thiamethoxam and spinosad on the survival and hypopharyngeal glands of the African honey bee (*Apis mellifera intermissa*). Entomol Gen 40:207–215 (2020).

[10] Stern VMRF , Smith R , Van den Bosch R and Hagen K , The integration of chemical and biological control of the spotted alfalfa aphid: the integrated control concept. Hilgardia 29:81–101 (1959).

[11] Heimpel GE and Mills NJ , Biological Control. Cambridge University Press, Cambridge, UK, p. 380 (2017).

[12] Rossi Stacconi MV , Amiresmaeili N , Biondi A , Carli C , Caruso S , Dindo ML *et al*., Biological control of the invasive spotted wing drosophila trough augmentative releases of the cosmopolitan parasitoid *Trichopria drosophilae* . Biol Control 117:188–196 (2018).

[13] Campos MR , Monticelli LS , Béarez P , Amiens‐Desneux E , Wang Y , Lavoir A‐V *et al*., Impact of a shared sugar food source on biological control of *Tuta absoluta* by the parasitoid *Necremnus tutae* . J Pest Sci 93:207–218 (2020).

[14] Santoiemma G , Tonina L , Marini L , Duso C and Mori N , Integrated management of *Drosophila suzukii* in sweet cherry orchards. Entomol Gen 40:297–305 (2020).

[15] SANCO/10329/2002 . Guidance Document on Terrestrial Ecotoxicology under Council Directive 91/414/EEC, Rev.2 Final, pp. 1–39 (2002).

[16] Barrett KL , Grandy N , Harrison EG , Hassan S and Oomen P , Guidance document on regulatory testing procedures for pesticides and non‐target arthropods. ESCORT workshop (European standard characteristics of non‐target arthropod regulatory testing). In Wageningen, the Netherlands: a Joint BART, EPPO/CoE and IOBC Workshop Organised in Conjunction with SETAC‐Europe and Funded by the EC, 51 p. (1994).

[17] EFSA Panel on Plant Protection Products and their Residues (PPR) , Scientific opinion addressing the state of the science on risk assessment of plant protection products for non‐target arthropods. EFSA J 13:3996 (2015).

[18] Desneux N , Fauvergue X , Dechaume‐Moncharmont FX , Kerhoas L , Ballanger Y and Kaiser L , *Diaeretiella rapae* limits *Myzus persicae* populations after applications of deltamethrin in oilseed rape. J Econ Entomol 98:9–17 (2005). 15765661 10.1093/jee/98.1.9

[19] Guedes RNC , Smagghe G , Stark JD and Desneux N , Pesticide‐induced stress in arthropod pests for optimized integrated pest management programs. Annu Rev Entomol 61:43–62 (2016). 26473315 10.1146/annurev-ento-010715-023646

[20] Langhof M , Gathmann A , Poehling HM and Meyhöfer R , Impact of insecticide drift on aphids and their parasitoids: residual toxicity, persistence and recolonisation. Agric Ecosyst Environ 94:265–274 (2003).

[21] Marubayashi RY , Oliveira RBD , Ferreira MDC , Roggia S , EDD M and Saab OJ , Insecticide spray drift reduction with different adjuvants and spray nozzles. Revista Brasileira de Engenharia Agrícola e Ambiental 25:282–287 (2021).

[22] Biondi A , Desneux N , Siscaro G and Zappalà L , Using organic‐certified rather than synthetic pesticides may not be safer for biological control agents: selectivity and side effects of 14 pesticides on the predator *Orius laevigatus* . Chemosphere 87:803–812 (2012). 22342338 10.1016/j.chemosphere.2011.12.082

[23] He Y , Zhao J , Zheng Y , Weng Q , Biondi A , Desneux N *et al*., Assessment of potential sublethal effects of various insecticides on key biological traits of the tobacco whitefly, *Bemisia tabaci* . Int J Biol Sci 9:246–255 (2013). 23494876 10.7150/ijbs.5762 PMC3596710

[24] Dai C , Ricupero M , Puglisi R , Lu Y , Desneux N , Biondi A *et al*., Can contamination by major systemic insecticides affect the voracity of the harlequin ladybird? Chemosphere 256:126986 (2020). 32445995 10.1016/j.chemosphere.2020.126986

[25] Passos LC , Soares MA , Collares LJ , Malagoli I , Desneux N and Carvalho GA , Lethal, sublethal and transgenerational effects of insecticides on *Macrolophus basicornis*, predator of *Tuta absoluta* . Entomol Gen 38:127–143 (2018).

[26] Soares MA , Campos MR , Passos LC , Carvalho GA , Haro MM , Lavoir AV *et al*., Botanical insecticide and natural enemies: a potential combination for pest management against *Tuta absoluta* . J Pest Sci 92:1433–1443 (2019).

[27] Arnó J and Gabarra R , Side effects of selected insecticides on the *Tuta absoluta* (Lepidoptera: Gelechiidae) predators *Macrolophus pygmaeus* and *Nesidiocoris tenuis* (Hemiptera: Miridae). J Pest Sci 84:513–520 (2011).

[28] Wanumen AC , Sánchez‐Ramos I , Viñuela E , Medina P and Adán Á , Impact of feeding on contaminated prey on the life parameters of *Nesidiocoris tenuis* (Hemiptera: Miridae) adults. J Insect Sci 16:1–7 (2016). 27694345 10.1093/jisesa/iew084 PMC5043474

[29] Jaworski CC , Bompard A , Genies L , Amiens‐Desneux E and Desneux N , Preference and prey switching in a generalist predator attacking local and invasive alien pests. PLoS One 8:1–10 (2013). 10.1371/journal.pone.0082231 PMC3846826 24312646

[30] Biondi A , Guedes RNC , Wan F‐H and Desneux N , Ecology, worldwide spread, and management of the invasive south American tomato pinworm, *Tuta absoluta*: past, present, and future. Annu Rev Entomol 63:239–258 (2018). 28977774 10.1146/annurev-ento-031616-034933

[31] Van Lenteren JC , Lanzoni A , Hemerik L , Bueno VHP , Bajonero Cuervo JG , Biondi A *et al*., The pest kill rate of thirteen natural enemies as aggregate evaluation criterion of their biological control potential of *Tuta absoluta* . Sci Rep 11:10756 (2021). 34031491 10.1038/s41598-021-90034-8 PMC8144571

[32] Desneux N , Han P , Mansour R , Arnó J , Brévault T , Campos MR *et al*., Integrated pest management of *Tuta absoluta*: practical implementations across different world regions. J Pest Sci 95:17–39 (2022).

[33] Zappalà L , Biondi A , Alma A , Al‐Jboory IJ , Arnò J , Bayram A *et al*., Natural enemies of the south American moth, *Tuta absoluta*, in Europe, North Africa and Middle East, and their potential use in pest control strategies. J Pest Sci 86:635–647 (2013).

[34] Jaworski CC , Chailleux A , Bearez P and Desneux N , Apparent competition between major pests reduces pest population densities on tomato crop, but not yield loss. J Pest Sci 88:793–803 (2015).

[35] Naselli M , Urbaneja A , Siscaro G , Jaques JA , Zappalà L , Flors V *et al*., Stage‐related defense response induction in tomato plants by *Nesidiocoris tenuis* . Int J Mol Sci 17:1210 (2016). 10.3390/ijms17081210 PMC5000608 27472328

[36] Mansour R , Brévault T , Chailleux A , Cherif A , Grissa‐Lebdi K , Haddi K *et al*., Occurrence, biology, natural enemies and management of *Tuta absoluta* in Africa. Entomol Gen 38:83–112 (2018).

[37] Thomine E , Jeavons E , Rusch A , Bearez P and Desneux N , Effect of crop diversity on predation activity and population dynamics of the mirid predator *Nesidiocoris tenuis* . J Pest Sci 93:1255–1265 (2020).

[38] Thomine E , Rusch A , Supplisson C , Monticelli LS , Amiens‐Desneux E , Lavoir AV *et al*., Highly diversified crop systems can promote the dispersal and foraging activity of the generalist predator *Harmonia axyridis* . Entomol Gen 40:133–145 (2020).

[39] Castañé C , Arnó J , Gabarra R and Alomar O , Plant damage to vegetable crops by zoophytophagous mirid predators. Biol Control 59:22–29 (2011).

[40] Mollá O , Biondi A , Alonso‐Valiente M and Urbaneja A , A comparative life history study of two mirid bugs preying on *Tuta absoluta* and *Ephestia kuehniella* eggs on tomato crops: implications for biological control. BioControl 59:175–183 (2014).

[41] Siscaro G , Pumo CL , Garzia GT , Tortorici S , Gugliuzzo A , Ricupero M *et al*., Temperature and tomato variety influence the development and the plant damage induced by the zoophytophagous mirid bug *Nesidiocoris tenuis* . J Pest Sci 92:1049–1056 (2019).

[42] Campos MR , Biondi A , Adiga A , Guedes RNC and Desneux N , From the Western Palaearctic region to beyond: *Tuta absoluta* ten years after invading Europe. J Pest Sci 90:787–796 (2017).

[43] Ferracini C , Bueno VH , Dindo ML , Ingegno BL , Luna MG , Salas Gervassio NG *et al*., Natural enemies of *Tuta absoluta* in the Mediterranean basin, Europe and South America. Biocontrol Sci Tech 29:578–609 (2019).

[44] Mansour R and Biondi A , Releasing natural enemies and applying microbial and botanical pesticides for managing *Tuta absoluta* in the MENA region. Phytoparasitica 49:179–194 (2021).

[45] Pérez‐Hedo M , Alonso‐Valiente M , Vacas S , Gallego C , Rambla JL , Navarro‐Llopis V *et al*., Eliciting tomato plant defenses by exposure to herbivore induced plant volatiles. Entomol Gen 41:209–218 (2021).

[46] Madbouni MAZ , Samih MA , Qureshi JA , Biondi A and Namvar P , Compatibility of insecticides and fungicides with the zoophytophagous mirid predator *Nesidiocoris tenuis* . PLoS One 12:e0187439 (2017). 29095873 10.1371/journal.pone.0187439 PMC5667899

[47] Soares MA , Carvalho GA , Campos MR , Passos LC , Haro MM , Lavoir AV *et al*., Detrimental sublethal effects hamper the effective use of natural and chemical pesticides in combination with a key natural enemy of *Bemisia tabaci* on tomato. Pest Manag Sci 76:3551–3559 (2020). 32452608 10.1002/ps.5927

[48] Biondi A , Zappalà L , Di Mauro A , Tropea Garzia G , Russo A , Desneux N *et al*., Can alternative host plant and prey affect phytophagy and biological control by the zoophytophagous mirid *Nesidiocoris tenuis*? BioControl 61:79–90 (2016).

[49] Tan Y , Biondi A , Desneux N and Gao XW , Assessment of physiological sublethal effects of imidacloprid on the mirid bug *Apolygus lucorum* (Meyer‐Dür). Ecotoxicology 21:1989–1997 (2012). 22740097 10.1007/s10646-012-0933-0

[50] Naselli M , Zappala L , Gugliuzzo A , Garzia GT , Biondi A , Rapisarda C *et al*., Olfactory response of the zoophytophagous mirid *Nesidiocoris tenuis* to tomato and alternative host plants. Arthropod‐Plant Interactions 11:121–131 (2017).

[51] Finney DJ , Probit Analysis; A Statistical Treatment of the Sigmoid Response Curve. Macmillan, Oxford, England, 256 p. (1947).

[52] Shapiro SS and Wilk MB , An analysis of variance test for normality (complete samples). Biometrika 52:591–611 (1965).

[53] Bartlett MS , Properties of sufficiency and statistical tests. Proc R Soc Lond A 160:268–282 (1937).

[54] Nelder JA and Wedderburn RWM , Generalized Linear Models. J Roy Statistic Soc 135:370–384 (1972).

[55] Fox J and Weisberg S , An R Companion to Applied Regression, Third edn. Sage, Thousand Oaks, CA (2019).

[56] Venables WN and Ripley BD , MASS: Modern Applied Statistics with S, Fourth edn. Springer, New York (2002).

[57] Hothorn T , Bretz F and Westfall P , Simultaneous inference in general parametric models. Biom J 50:346–363 (2008). 18481363 10.1002/bimj.200810425

[58] Yu SJ , The Toxicology and Biochemistry of Insecticides. CRC Press, Boca Raton, FL, p. 276 (2008).

[59] Casida JE and Durkin KA , Neuroactive insecticides: targets, selectivity, resistance, and secondary effects. Annu Rev Entomol 58:99–117 (2013). 23317040 10.1146/annurev-ento-120811-153645

[60] Carvalho GA , Grützmacher AD , Passos LC and Oliveira RL , Physiological and ecological selectivity of pesticides for natural enemies of insects, in Natural Enemies of Insect Pests in Neotropical Agroecosystems, ed. by Souza B , Vásquez L and Marucci R . Springer, Cham, Switzerland, pp. 469–478 (2019).

[61] Torres JB and Bueno ADF , Conservation biological control using selective insecticides – a valuable tool for IPM. Biol Control 126:53–64 (2018).

[62] Mollá O , González‐Cabrera J and Urbaneja A , The combined use of *bacillus thuringiensis* and *Nesidiocoris tenuis* against the tomato borer *Tuta absoluta* . BioControl 56:883–891 (2011).

[63] Desneux N , Pham‐Delègue M and Kaiser L , Effects of sub‐lethal and lethal doses of lambda‐cyhalothrin on oviposition experience and host‐searching behaviour of a parasitic wasp, *Aphidius ervi* . Pest Manag Sci 60:381–389 (2004). 15119601 10.1002/ps.822

[64] Eijaza S , Khan MF , Mahmood K , Anwar M , Alamgir A and Khatri I , Studies on degradation and efficacy of synthetic pesticides on okra crop. Acad J Entomol 8:12–18 (2015).

[65] Ricupero M , Desneux N , Zappalà L and Biondi A , Target and non‐target impact of systemic insecticides on a polyphagous aphid pest and its parasitoid. Chemosphere 247:125728 (2020). 32069706 10.1016/j.chemosphere.2019.125728

[66] Wang R , Zheng H , Qu C , Wang Z , Kong Z and Luo C , Lethal and sublethal effects of a novel cis‐nitromethylene neonicotinoid insecticide, cycloxaprid, on *Bemisia tabaci* . Crop Prot 83:15–19 (2016).

[67] Fernandes ME , Alves FM , Pereira RC , Aquino LA , Fernandes FL and Zanuncio JC , Lethal and sublethal effects of seven insecticides on three beneficial insects in laboratory assays and field trials. Chemosphere 156:45–55 (2016). 27160634 10.1016/j.chemosphere.2016.04.115

[68] Wang S , Qi Y , Desneux N , Shi X , Biondi A and Gao X , Sublethal and transgenerational effects of short‐term and chronic exposures to the neonicotinoid nitenpyram on the cotton aphid *Aphis gossypii* . J Pest Sci 90:389–396 (2017).

[69] Vojoudi S , Saber M , Hejazi MJ and Talaei‐Hassanloui R , Toxicity of chlorpyrifos, spinosad and abamectin on cotton bollworm, *Helicoverpa armigera* and their sublethal effects on fecundity and longevity. Bull Insectol 64:189–193 (2011).

[70] Messing R and Croft BA , Sublethal influences, in Arthropod Biological Control Agents and Pesticides, ed. by Croft BA . Jon Willey and Sons, New York, pp. 157–183 (1990).

[71] Biondi A , Zappala L , Desneux N , Aparo A , Siscaro G , Rapisarda C *et al*., Potential toxicity of α‐cypermethrin‐treated nets on *Tuta absoluta* (Lepidoptera: Gelechiidae). J Econ Entomol 108:1191–1197 (2015). 26470245 10.1093/jee/tov045

[72] Gholamzadeh‐Chitgar M , Hajizadeh J , Ghadamyari M , Karimi‐Malati A and Hoda H , Effects of sublethal concentration of diazinon, fenitrothion and chlorpyrifos on demographic and some biochemical parameters of predatory bug, *Andrallus spinidens* Fabricius (Hemiptera: Pentatomidae) in laboratory conditions. Int J Pest Manag 61:204–211 (2015).

[73] Ricupero M , Abbes K , Haddi K , Kurtulus A , Desneux N , Russo A *et al*., Combined thermal and insecticidal stresses on the generalist predator *Macrolophus pygmaeus* . Sci Total Environ 729:138922 (2020). 32498167 10.1016/j.scitotenv.2020.138922

[74] Kim DS , Brooks DJ and Riedl H , Lethal and sublethal effects of abamectin, spinosad, methoxyfenozide and acetamiprid on the predaceous plant bug *Deraeocoris brevi*s in the laboratory. Biocontrol 51:465–484 (2006).

[75] Biondi A , Mommaerts V , Smagghe G , Viñuela E , Zappalà L and Desneux N , The non‐target impact of spinosyns on beneficial arthropods. Pest Manag Sci 68:1523–1536 (2012). 23109262 10.1002/ps.3396

[76] Khambay BPS and Jewess PJ , Pyrethroids, in Insect Control – Biological and Synthetic Agents, ed. by Gilbert LI and Gill SS . Academic Press, London, UK, pp. 1–29 (2010).

[77] D’Ávila VA , Barbosa WF , Guedes RN and Cutler GC , Effects of spinosad, imidacloprid, and lambda‐cyhalothrin on survival, parasitism, and reproduction of the aphid parasitoid *Aphidius colemani* . J Econ Entomol 111:1096–1103 (2018). 29528456 10.1093/jee/toy055

[78] Sharifian I , Sabahi Q and Bandani AR , Effect of some conventional insecticides on functional response parameters of *Macrolophus pygmaeus* (hem.: Miridae) on *Tuta absoluta* (Lep.: Gelechiidae). Bihar Biol 11:10–14 (2017).

[79] Barbosa WF , Tomé HV , Bernardes RC , Siqueira MA , Smagghe G and Guedes RN , Biopesticide‐induced behavioral and morphological alterations in the stingless bee *Melipona quadrifasciata* . Environ Toxicol Chem 34:2149–2158 (2015). 26190792 10.1002/etc.3053

[80] Crouse GD , Sparks TC , Schoonover J , Gifford J , Dripps J , Bruce T *et al*., Recent advances in the chemistry of spinosyns. Pest Manag Sci 57:177–185 (2011). 10.1002/1526-4998(200102)57:2<177::AID-PS281>3.0.CO;2-Z 11455648

[81] Sparks TC , Crouse GD and Durst G , Natural products as insecticides: the biology, biochemistry and quantitative structure‐activity relationships of spinosyns and spinosoids. Pest Manag Sci 57:896–905 (2001). 11695182 10.1002/ps.358

[82] Salgado VL and Sparks TC , The Spinosins: chemistry, biochemistry, mode of action, and resistance, in Insect Control – Biological and Synthetic Agents, ed. by Gilbert LI and Gill SS . Academic Press, London, UK, pp. 207–243 (2010).

[83] Zhang X , Xu Q , Lu W and Liu F , Sublethal effects of four synthetic insecticides on the generalist predator *Cyrtorhinus lividipennis* . J Pest Sci 88:383–392 (2015).

[84] El‐Arnaouty SA , Galal HH , Afifi V , Beyssat J , Pizzol J , Desneux N *et al*., Assessment of two Trichogramma species for the control of *Tuta absoluta* in north African tomato greenhouses. Afric Entomol 22:801–809 (2014).

[85] Salas Gervassio NG , Aquino D , Vallina C , Biondi A and Luna MG , A re‐examination of *Tuta absoluta* parasitoids in South America for optimized biological control. J Pest Sci 92:1343–1357 (2019).

[86] Ingegno BL , La‐Spina M , Jordan MJ , Tavella L and Sanchez JA , Host plant perception and selection in the sibling species *Macrolophus melanotoma* and *Macrolophus pygmaeus* (Hemiptera: Miridae). J Insect Behav 29:117–142 (2016).

[87] Zhang R , Gao G and Chen H , Silencing of the olfactory co‐receptor gene in *Dendroctonus armandi* leads to EAG response declining to major host volatiles. Sci Rep 6:23136 (2016). 26979566 10.1038/srep23136 PMC4793246

[88] Wang GY , Zhu MF , Jiang YD , Zhou WW , Liu S , Heong KL *et al*., Identification of candidate odorant‐binding protein and chemosensory protein genes in *Cyrtorhinus lividipennis* (Hemiptera: Miridae), a key predator of the rice planthoppers in Asia. Environ Entomol 46:654–662 (2017). 28407047 10.1093/ee/nvx075

